# Sequential Photoperiodic Programing of Serotonin Neurons, Signaling and Behaviors During Prenatal and Postnatal Development

**DOI:** 10.3389/fnins.2019.00459

**Published:** 2019-05-08

**Authors:** Justin K. Siemann, Noah H. Green, Nikhil Reddy, Douglas G. McMahon

**Affiliations:** ^1^Department of Biological Sciences, Vanderbilt University, Nashville, TN, United States; ^2^Silvio O. Conte Center for Neuroscience Research, Vanderbilt University, Nashville, TN, United States; ^3^Vanderbilt Undergraduate Neuroscience Program, Vanderbilt University, Nashville, TN, United States; ^4^Department of Pharmacology, Vanderbilt University, Nashville, TN, United States; ^5^Kennedy Center for Research on Human Development, Vanderbilt University, Nashville, TN, United States

**Keywords:** photoperiod, development, serotonin, dorsal raphe, depression, anxiety

## Abstract

Early life stimuli during critical developmental time frames have been linked to increased risk for neurodevelopmental disorders later in life. The serotonergic system of the brain is implicated in mood disorders and is impacted by the duration of daylight, or photoperiod. Here we sought to investigate sensitive periods of prenatal and postnatal development for photoperiodic programming of DRN serotonin neurons, midbrain serotonin and metabolite levels along with affective behaviors in adolescence (P30) or adulthood (P50). To address these questions we restricted the interval of exposure to prenatal development (E0-P0) for Long summer-like photoperiods (LD 16:8), or Short winter-like photoperiods (LD 8:16) with postnatal development and maturation then occurring under the opposing photoperiod. Prenatal exposure alone to Long photoperiods was sufficient to fully program increased excitability of DRN serotonin neurons into adolescence and adulthood, similar to maintained exposure to Long photoperiods throughout development. Interestingly, Long photoperiod exposure can elevate serotonin and its’ corresponding metabolite levels along with reducing affective behavior, which appear to have both pre and postnatal origins. Thus, exposure to Long photoperiods prenatally programs increased DRN serotonin neuronal excitability, but this step is insufficient to program serotonin signaling and affective behavior. Continuing influence of Long photoperiods during postnatal development then modulates serotonergic content and has protective effects for depressive-like behavior. Photoperiodic programing of serotonin function in mice appears to be a sequential process with programing of neuronal excitability as a first step occurring prenatally, while programing of circuit level serotonin signaling and behavior extends into the postnatal period.

## Introduction

Exposure to environmental factors during key neurodevelopmental time points such as gestation and postnatal development have been associated with increased risk for psychiatric disorders later in life (Nestler et al., 2002; Andersen, 2015). The duration of daylight or photoperiod has been implicated as a risk factor for neurodevelopmental disorders (Modai et al., 1994; Castrogiovanni et al., 1998; Chotai et al., 2003). Monoamine turnover of serotonin and dopamine is lower during winter and fall seasons (Chotai and Adolfsson, 2002; Lambert et al., 2002) and an interaction between candidate genes for mood disorders and births in winter/fall seasons has been identified, demonstrating a gene × environment risk for these disorders (Chotai et al., 2003). Most recently, human epidemiological work suggests that high amplitude photoperiodic changes during the second trimester of gestation can result in decreased risk for depression in the offspring later in life (Devore et al., 2018). Thus, the day length or photoperiod influences the development of affective disorders.

The serotonergic system is impacted by the duration of daylight, and has been consistently implicated in mood disorders (Uher and McGuffin, 2008; Ciarleglio et al., 2011b; Spindelegger et al., 2012). A main hub for serotonin (5-HT) signaling in the brain is the dorsal raphe nucleus (DRN) (Gaspar et al., 2003) and abnormal serotonin signaling during prenatal and perinatal development results in dramatic and lasting changes in adulthood at the molecular, circuit, and behavioral levels (Gingrich and Hen, 2001; Bennett et al., 2002; Gaspar et al., 2003; Bonnin et al., 2011; Bonnin and Levitt, 2011). Knockout animal models targeting molecular components of the serotonergic system exhibit altered 5-HT neuronal development and viability (Azmitia, 2001; Hendricks et al., 2003; Persico et al., 2003), circuit formation and monoamine content (Bennett-Clarke et al., 1993; Cases et al., 1995, 1996; Upton et al., 1999; Richardson-Jones et al., 2011; Migliarini et al., 2013) and affective behaviors (Gross et al., 2002; Holmes et al., 2002, 2003; Kalueff et al., 2007). In addition, environmental signals during development including photoperiod, impact monoamine content as well as depressive-like behaviors in preclinical models of mood disorders (Anisman and Matheson, 2005; Green et al., 2015; Qin et al., 2015; Xu et al., 2016). Specifically, adult mice that have been exposed both prenatally and postnatally to Long summer-like photoperiods demonstrate increased excitability of dorsal raphe serotonin neurons (Green et al., 2015), increased midbrain serotonin and norepinephrine concentrations (Otsuka et al., 2014; Goda et al., 2015; Green et al., 2015) and decreased depressive-like and anxiety-like behaviors, compared to animals developed and matured under Short winter-like photoperiods (Prendergast and Nelson, 2005; Pyter and Nelson, 2006; Otsuka et al., 2014; Green et al., 2015). Importantly, developmental photoperiod exposure was shown to drive the increased firing rate of dorsal raphe serotonin neurons in adulthood (Green et al., 2015). These findings suggest that there are sensitive periods during development during which photoperiod acts to program the serotonergic system relevant to the pathology of mood disorders.

In the current study we sought to evaluate when during development do Long summer-like photoperiods impact serotonin neuronal firing rate, midbrain serotonergic content, and depressive and anxiety-like behaviors, as well as what stage in maturation, adolescence or adulthood, are photoperiod programing effects apparent. Here, we used a photoperiod paradigm with high amplitude changes during gestation following Devore et al., who investigated how high photoperiod amplitudes during key prenatal periods of human development impacted the risk for depression later in life. To test this, we exposed mice to Long summer-like photoperiods during gestation *in utero*, then exposed mice to Short winter-like photoperiods beginning at birth and continued through postnatal development and maturation (L-S), or exposed mice to Short winter-like photoperiods gestationally, and then switched mice to Long summer-like photoperiods at birth (S-L). We assayed DRN neuronal firing rate, serotonin and its’ metabolites, and depressive and anxiety-like behaviors at postnatal days 30 and 50, representing early adolescence and early adulthood, respectively, in mice (Hefner and Holmes, 2007), in order to assess for enduring effects of photoperiod exposure during development.

## Materials and Methods

**FIGURE 1:**
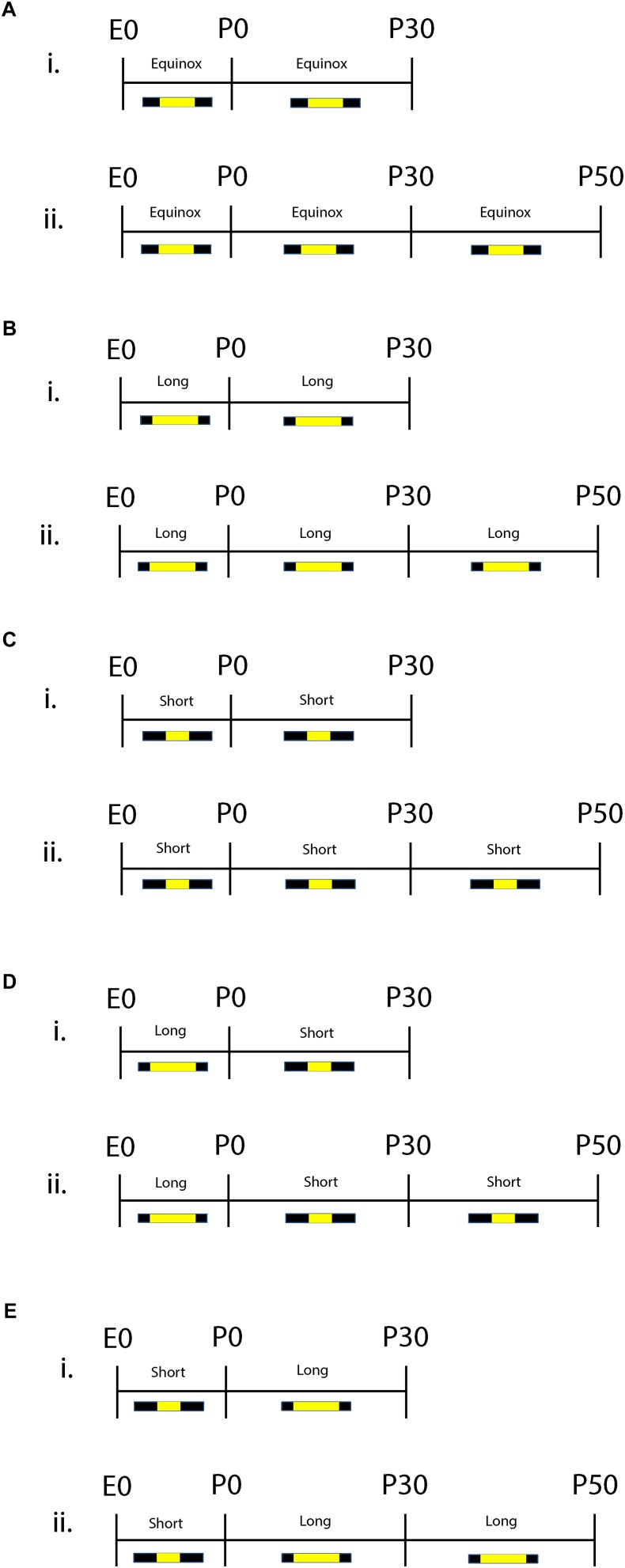
Schematic of developmental photoperiod paradigm. Animals were maintained on Equinox (Eq) **(A)**, Long (L) (**B),** or Short (S) **(C)** photoperiods from E0 to P30 or P50. One cohort of animals developed from E0 under Long photoperiods and were switched to Short photoperiods (L-S) at birth (P0) (**D).** Another cohort of animals were developed from E0 under Short photoperiods and were switched to a Long photoperiod at birth (S-L) (**E).** Experiments were performed at two different time points: P30 **(i)** or P50 **(ii)**. Photoperiods were defined as follows: Equinox, LD 12:12; Long, LD 16:8; Short, LD 8:16 where L, light; D, dark (i.e., LD 8:16 means Light for 8 h, Dark for 16 h).

Both male and female C3Hf^+/+^ mice were group housed and used for the studies. C3Hf^+/+^ mice were used because they are genetically competent to synthesize the key season hormone melatonin, unlike many mouse strains that harbor mutations in the melatonin synthesis pathway (Ebihara et al., 1986, 1987) and do not contain the retinal degeneration alleles present in the parent C3H strain (Schmidt and Lolley, 1973). The experimental paradigm is shown in Figure 1. Mice developed and were raised under Equinox (i.e., Eq) (12 h of light 12 h of darkness), Short (i.e., S) (8 h of light and 16 h of darkness), or Long (i.e., L) (16 h of light and 8 h of darkness) photoperiods. For sensitive period experiments, animals developed under either Long photoperiods from embryonic day 0 (E0) and were switched at birth to Short photoperiods (i.e., L-S, postnatal day 0 = P0) or developed under Short photoperiods from E0 and were switched at birth to Long photoperiods (i.e., S-L). Experiments were performed at two different developmental time points: P30 (ranging from P30 to P40) and at P50 (ranging from P50 to P90) and were conducted near the midday of each light cycle. The light intensity for each light cycle was measured at ∼1,000 lux. All experiments performed were in accordance with the Vanderbilt University Institutional Animal Care and Use Committee and National Institutes of Health guidelines.

### Multielectrode Array Electrophysiological Recording

Mouse brains (*n* = 4–7 per experimental group) were collected near the midday of each light cycle and placed in cold, oxygenated (95%O_2_–5%CO_2_) dissecting media (in mM: 114.5 NaCl, 3.5 KCl, 1 NaH2PO4, 1.3 MgSO4, 2.5 CaCl2, 10 d(+)-glucose, and 35.7 NaCHO3). 300 μm thick coronal slices were taken between −5.5 and −5.75 mm from Bregma using a Vibrotome (Leica Biosystems). DRN tissue was isolated and then placed into room temperature, oxygenated (95%O_2_–5%CO_2_), extracellular recording media (in mM: 124 NaCl, 3.5 KCl, 1 NaH2PO4, 1.3 MgSO4, 2.5 CaCl2, 10 d(+)-glucose, and 26 NaHCO3).

Dorsal raphe nucleus slices of 300 micron thickness were placed on multi electrode arrays, electrophysiology harps were used to restrain DRN slices and 40 μM tryptophan and 3 μM phenylephrine were added to the recording solution and perfused at a rate of 1.3 mL/min over the DRN slice once the recording began. 8OH-DPAT, at a concentration of 1 μM, was used to identify dorsal raphe 5-HT neurons by eliciting 5-HT1a-mediated suppression of spontaneous firing rate and was perfused at a rate of 1.3 mL/min over the slice for 5–6 min following 4–5 min of recording with recording solution. 6 × 10 perforated arrays (Multi Channel Systems) were utilized to measure 5-HT neuronal firing rate in the ventromedial portion of the dorsal raphe nucleus. The diameter of the electrodes were 30 microns with 100 micron spacing in between each electrode resulting in the electrodes covering an area of 1200 microns below the cerebral aqueduct and 340 microns laterally on each side covering a total of 680 micron width (Supplementary Figure S1).

### Monoamine Analysis

Mouse mid-brains (*n* = 12–14 per group) were removed near midday and midbrain sections were dissected with clean razor blades. 1.5 mm biopsy punches (Integra Miltex) were used to target the dorsal raphe region from these sections and the tissue was placed in 1.5 mL tubes and then frozen in liquid nitrogen. Biogenic amine analysis was performed in the Vanderbilt Neurochemistry Core Laboratory. Briefly, samples were stored at −80°C, then the tissue was homogenized with a tissue dismembrator, in 100–750 μl of 0.1 M TCA. This contained 10–2 M sodium acetate, 10–4 M EDTA, 5ng/mL isoproterenol (as internal standard) and 10.5% methanol (pH 3.8). Samples were then spun in a microcentrifuge at 10000×*g* for 20 min with the supernatant being removed and stored at –80°C and the pellet were saved for protein analysis. Supernatant was then thawed and spun for 20 min and the samples of the supernatant were analyzed for biogenic amines. These amines were determined by a specific HPLC assay utilizing an Antec Decade II (oxidation: 0.4) (3 mm GC WE, HYREF) electrochemical detector operated at 33°C. Twenty μl samples of the supernatant were injected using a Water 2707 autosampler onto a Phenomenex Kintex (2.6u, 100A) C18 HPLC column (100 × 4.60 mm), biogenic amines were eluted with a mobile phase consisting of 89.5% 0.1 M TCA, 10–2 M sodium acetate, 10-4 M EDTA and 10.5% methanol (pH 3.8) and the solvent was then delivered at 0.6 mL/min using a Waters 515 HPLC pump. Utilizing this HPLC solvent the following biogenic amines eluted were: Noradrenaline, Adrenaline, DOPAC, Dopamine, 5-HIAA, HVA, 5-HT, and 3-MT and HPLC control and data acquisition were managed by Empower software. Methods were the same as those previously published (Green et al., 2015).

### Behavioral Testing

Mice (*n* = 14–23 per group) were group housed and transferred from the housing facility to light tight boxes housed in the Vanderbilt Murine Neurobehavioral Core. Mice acclimated for 1 week in the new facility and then behavior on the open field test was assessed followed by the tail suspension test with each test conducted on successive days. All animals were tested in the middle of their light phase (1030–1500) and only two groups (L-S at P50 and Eq at P30) started earlier at 0930 and 1000, respectively, for the open field test due to scheduling constraints in the Vanderbilt Neurobehavioral Core. Mice underwent one test daily and did not perform the same test more than once. Pseudo-randomization in the order of testing did not occur and male mice were tested before female mice. Therefore, all the male mice in one cage were tested in succession and once every male mouse was run through the behavioral task then the cages of female mice were tested. Mice acclimated in the testing rooms for at least 30 min prior to each test.

### Open Field Test

Locomotor activity was measured in an open field chamber measuring 27 cm × 27 cm, with light intensities ranging between 50 and 135 lux across all chambers during the 60-min test period. Thigmotaxis represented the amount of time spent in the outer area of the chamber, which was defined as the area 4.25 cm from the wall and represents 50% of the total chamber area. Total thigmotaxis was measured during the 60-min test in 5-min blocks and was calculated from all 12 blocks within 60 min of testing. Data was obtained with (Med Associates Inc., St. Albans, VT, United States) open field software.

### Tail Suspension Test

Each mouse had its’ tail taped to a force meter and was suspended for entirety of the 6-min test. Total time spent immobile was measured through the force meter with a threshold of 7 (arbitrary units) and gain of 8 (arbitrary units) as the limit for struggling. Total time spent immobile was calculated throughout the 6 min test in 1 min blocks. Data was obtained with Med Associates Inc., tail suspension software and testing occurred under normal lighting conditions.

### Statistical Analysis

#### Electrophysiology Experiments

A Bessel filter with a 150 Hz frequency cut off was applied to raw data traces in Offline Sorter (Plexon Inc.). Detection threshold was manually set to include all spikes as well as the least amount of unipolar noise, which ranged from 13 to 35 μV. After detection of spikes occurred, these were then sorted by a K means scan method. The K means scan divided spikes into groups based on amplitude, power under the curve and spike duration and then manual verification was used to further identify legitimate spikes. Waveforms were sorted into groups, determined to be biologically relevant and were then validated by eye and spikes that did not fit the average waveform shape were then excluded from analysis. The total number of spikes was calculated during the recording before the application of 8-OH-DPAT. The number of spikes was then divided by the total time when those spikes occurred during the application of slice solution resulting in a measure of spikes per second. 8-OH-DPAT was applied to the recording solution for 5–6 min in order to determine and identify 5-HT neurons via feedback inhibition. Cells that demonstrated at least 50% suppression in the spike rate during 8-OH-DPAT application were then included in our analyses. Spontaneous firing rate data used for Eq (*n* = 6 mice, 92 cells), Short (*n* = 6 mice, 70 cells) and Long (*n* = 6 mice, 153 cells) groups at P50 were previously published in Green et al., 2015 Curr Biology.

#### Monoamine Concentration and Behavior Experiments

Prism 7 (Graphpad Software Inc., La Jolla, CA, United States) was used for all statistical analyses. Statistical significance was determined by two-way ANOVAs with a *p*-value less than 0.05 considered significant, all *post hoc* analysis Holm-Sidak’s multiple comparison tests were performed and standard error of the mean was used for all experiments unless otherwise specified. D’Agostino and Pearson normality tests were used and any mouse that was outside the normal distribution for any test was excluded from analysis (Tatem et al., 2014) and final group sizes are described in the Supplementary Methods.

## Results

### Prenatal Long Photoperiod Exposure Programs 5-HT Neuron Excitability

To test for the lasting effects of prenatal photoperiod exposure on DRN serotonin neuron activity, mice were maintained on Long photoperiods from E0 to birth (P0) and then transferred to Short photoperiods until electrophysiological assays were performed at P30 or P50 (L-S cohort, Figure 1D), or exposed to Short photoperiods during gestation and transferred to Long photoperiods at birth and maintained there until assayed at P30 or P50 (S-L cohort, Figure 1E). Mice maintained on Equinox photoperiods from E0 to P30 or P50 served as controls (Eq cohort, Figure 1A). Lastly, we also evaluated the effects on DRN 5-HT neuronal firing rate for exposures to Long (L, Figure 1B) or Short (S, Figure 1C) photoperiods throughout gestation, postnatal development, and maturation.

**FIGURE 2:**
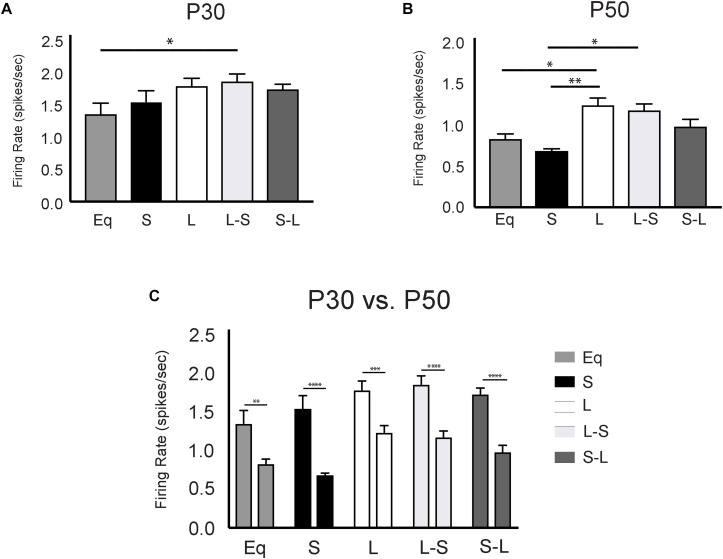
Long prenatal photoperiod programs lasting changes in firing rate of dorsal raphe serotonin (5-HT) neurons. **(A)** Analysis of 5-HT neuronal firing rate across photoperiods at P30, **(B)** at P50 and **(C)** within photoperiods during the P30 and P50 time points. Eq, equinox photoperiod; S, short photoperiod; L, long photoperiod; L-S, animals switched from a Long to Short photoperiod at birth and S-L represents animals switched from a Short to Long photoperiod at birth. The significant levels are as follows: (*p < 0.05, **p < 0.01, ***p < 0.001, ****p < 0.0001) using Holm-Sidak’s multiple comparison tests. At P30 we observed DRN firing rates for the following groups: Eq = 1.36 Hz +/– 0.16, *n* = 4 mice, 35 cells; *L* = 1.79 Hz +/– 0.12, *n* = 6 mice, 59 cells; *S* = 1.54 Hz +/– 0.17, *n* = 6 mice; 84 cells; L-S = 1.86 Hz +/– 0.11, *n* = 6 mice, 80 cells; S-L = 1.74 Hz +/– 0.08, *n* = 5 mice, 112 cells. In addition, at the P50 time point 5-HT neuronal firing rates were observed as: Eq = 0.83Hz +/– 0.06, *n* = 6 mice, 92 cells; *L* = 1.24 +/– 0.096, *n* = 6 mice, 153 cells; *S* = 0.68 Hz +/– 0.04, *n* = 6 mice, 70 cells; L-S = 1.18 Hz +/– 0.08, *n* = 5 mice, 30 cells; and S-L = 0.99 Hz +/– 0.08, *n* = 7 mice, 57 cells. Data used for the Eq, S, and L groups at the P50 time point were previously published in Green et al., 2015 Curr Biology.

Intriguingly, the 5-HT neuron firing rate from adult animals (P50) exposed to Long photoperiods only during gestation (L-S) was comparable to those exposed continuously to Long photoperiods throughout gestation, postnatal development, and maturation; both of which were significantly elevated compared to those exposed continuously to Short photoperiods throughout gestation, postnatal development, and maturation (Figure 2B). This further suggests that exposure to Long photoperiods during gestation is sufficient to program elevated DRN 5-HT neuron excitability into adulthood. In contrast, the 5-HT neuronal firing rate for animals exposed to Short photoperiods only during gestation (S-L) exhibited an intermediate effect. These spike rates were not statistically distinguishable, when using Holm-Sidak’s multiple comparison tests, from those exposed to Short photoperiods or to Long photoperiods throughout gestation, postnatal development, and maturation (L vs. S-L, *p* = 0.2914, S vs. S-L, *p* = 0.1833, Figure 2B) suggesting that gestational exposure to Short photoperiods may prolong the sensitive period for programing excitability into early postnatal development. Utilizing a two-way ANOVA, a significant main effect of photoperiod (*p* < 0.0001; *F*(4, 47) = 7.921) and a significant main effect of age (*p* < 0.0001; *F*(1, 47) = 105.1) were found. At the P30 time point, using Holm-Sidak’s multiple comparison tests, significant differences were observed between L-S and Eq (*p* = 0.0252) photoperiods and a trend level effect was found between L and Eq (*p* = 0.0782) conditions (Figure 2A). In addition, at the P50 time point there were significant differences between the L-S and S (*p* = 0.0153) photoperiods and significant differences between L and Eq (*p* = 0.0459) and L and S (*p* = 0.0028) conditions (Figure 2B). Lastly, there were significant overall age effects with DRN 5-HT neuron firing rate increased at the P30 compared to the P50 time point. Significant age effect differences were observed for Eq (*p* = 0.0018), S (*p* < 0.0001), L (*p* = 0.0007), L-S (*p* < 0.0001), and S-L (*p* < 0.0001) photoperiods (Figure 2C).

### Long Pre and Postnatal Photoperiod Impacts Midbrain Serotonergic Content

**FIGURE 3:**
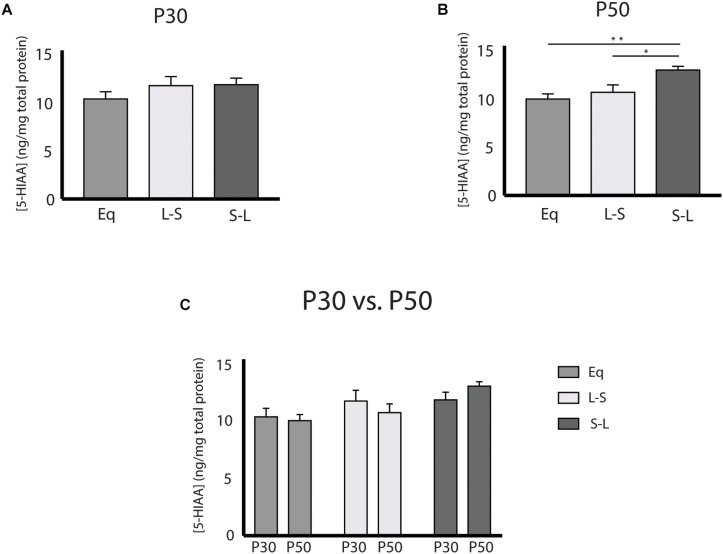
Postnatal Long photoperiod exposures result in lasting changes in 5-hydroxyindoleacetic Acid (5-HIAA) concentration. **(A)** Analysis of 5-HIAA content across photoperiods at P30, **(B)** at P50 and **(C)** within photoperiods during the P30 and P50 time points. The significance levels are as follows: (*p < 0.05, **p < 0.01) using Holm-Sidak’s multiple comparison tests.

**FIGURE 4:**
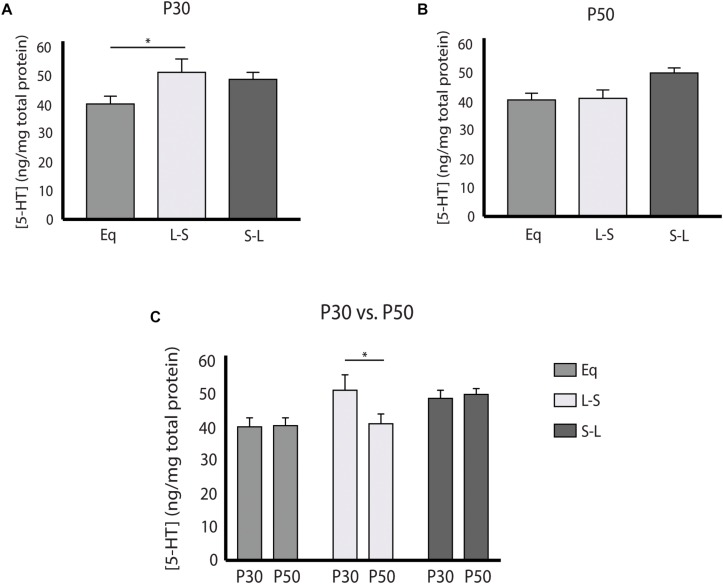
Pre and Postnatal Long photoperiod exposures impact serotonin (5-HT) concentration during development. **(A)** Analysis of 5-HT content across photoperiods at P30, **(B)** at P50, and **(C)** within photoperiods during the P30 and P50 time points. The significance levels are as follows: (*p < 0.05) using Holm-Sidak’s multiple comparison tests. C) When evaluating 5-HT levels within photoperiod, significant differences were observed between the P30 and P50 time points for the L-S photoperiod (*p* = 0.0433).

Our electrophysiology findings demonstrated that photoperiodic programming of DRN 5-HT neuron excitability can occur prenatally in response to Long photoperiods. We next focused on whether there was also a sensitive period for 5-HT content in the midbrain utilizing Equinox (Figure 1A), L-S (Figure 1D) and S-L (Figure 1E) photoperiod groups assayed at the P30 and P50 time points. Utilizing a two-way ANOVA, a significant main effect of photoperiod (*p* = 0.0053; *F*(2, 69) = 5.661) and a non-significant main effect of age (*p* = 0.9187; *F*(1, 69) = 0.0105) were observed (Figure 3C). At P50, significant increases in 5-HIAA, were observed for animals exposed to Long photoperiods postnatally (S-L vs. Eq, *p* = 0.0065; S-L vs. L-S, *p* = 0.0422) (Figure 3B). At P30, no significant effects were found (Figure 3A). Interestingly, we found evidence for both prenatal and postnatal programing of serotonin signaling in the midbrain. A significant main effect of photoperiod (*p* = 0.0082; *F*(2, 69) = 5.157) and a non-significant main effect of age (*p* = 0.2257; *F*(1, 69) = 1.495) were found by two-way ANOVA (Figure 4C). In adolescence (P30), Holm-Sidak’s multiple comparison tests revealed that 5-HT levels were significantly elevated in animals that were exposed to Long photoperiods gestationally (L-S vs. Eq, *p* = 0.0229), with a trend level increase in 5-HT levels in animals exposed to Long photoperiods after birth (S-L vs. Eq, *p* = 0.0715) (Figure 4A). In adulthood (P50), there were trend level increases for 5-HT in animals exposed to Long photoperiods postnatally (S-L vs. Eq, *p* = 0.0678, S-L vs. L-S, *p* = 0.0781), however, these values did not reach statistical significance (Figure 4B).

### Long Postnatal Photoperiod Exposure Results in Changes to Affective Behaviors

**FIGURE 5:**
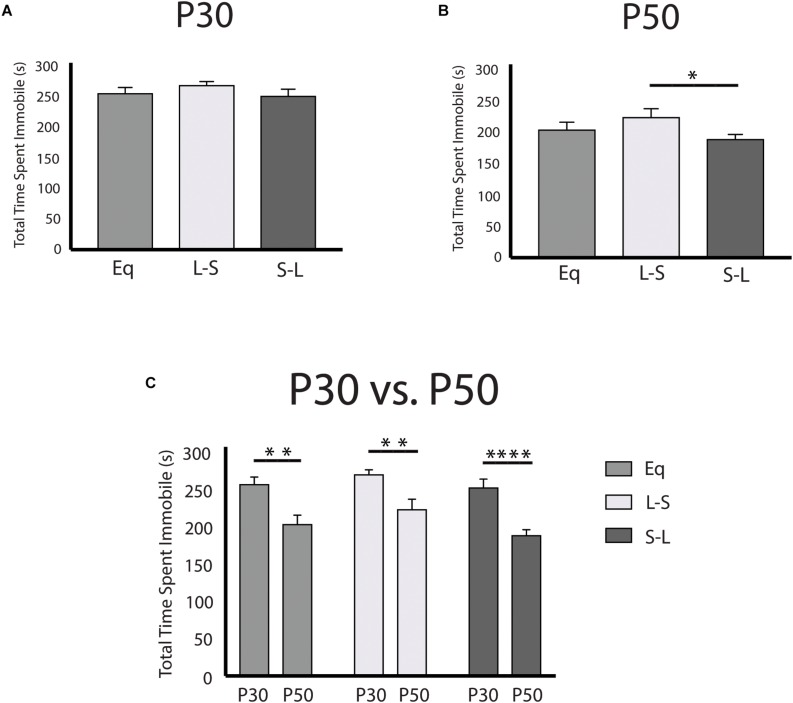
Postnatal Long photoperiod reduces depressive-like behavior in adulthood utilizing the tail suspension test. **(A)** Analysis of total time spent immobile across photoperiods at P30, **(B)** at P50, and **(C)** within photoperiods during the P30 and P50 time points. The significance levels are as follows: (*p < 0.05, **p < 0.01, ****p < 0.0001) using Holm-Sidak’s multiple comparison tests.

Having examined the prenatal programing of 5-HT neuron excitability, and the pre- and postnatal effects on midbrain serotonergic signaling, we next investigated the sensitive periods for photoperiodic effects on depressive and anxiety-like behaviors using the tail suspension test (TST) and the open field test (OFT), respectively. Overall, it was found that animals exposed to Long photoperiods postnatally demonstrate decreased depressive-like behavior in adulthood (Figure 5B) with these findings being driven by age (Figure 5C) and sex dependent effects (Supplementary Figure S2). A significant main effect of photoperiod (*p* = 0.0287; *F*(2, 94) = 3.688) and a significant main effect of age (*p* < 0.0001; *F*(1, 94) = 42.47) were found in the TST when evaluating the total time spent immobile. No significant effects were observed for P30 animals (Figure 5A). Adult animals (P50) that had been exposed to Long photoperiods after birth demonstrated less immobility than those that were exposed to Long photoperiods prior to birth (S-L vs. L-S, *p* = 0.0474) (Figure 5B). Lastly, time spent immobile was greater at P30 vs. P50 with significant age effects observed in *post hoc* tests for all groups (Eq, *p* = 0.0014; L-S, *p* = 0.0014; S-L, *p* < 0.0001) (Figure 5C). In addition, we found that postnatal photoperiod can impact anxiety-like behavior (Supplementary Figure S3). It was found that total thigmotaxis (i.e., time spent in the surround) was decreased for animals exposed to Long photoperiods during postnatal development. Using a two-way ANOVA, a significant main effect of photoperiod (*p* = 0.0114; *F*(2, 94) = 4.696) and a significant main effect of age (*p* < 0.0001; *F*(1, 94) = 16.73) were found when measuring total thigmotaxis (Supplementary Figure S3C). In addition, Long photoperiod exposure during postnatal development (S-L) resulted in trend level decreases of thigmotaxis, in adolescence (P30) (Supplementary Figure S3A) and in adulthood (P50) (Supplementary Figure S3B). Significant age effects again were observed with adolescent (P30) animals demonstrating increased total thigmotaxis compared to adult (P50) mice (Supplementary Figure S3C).

## Discussion

**FIGURE 6:**
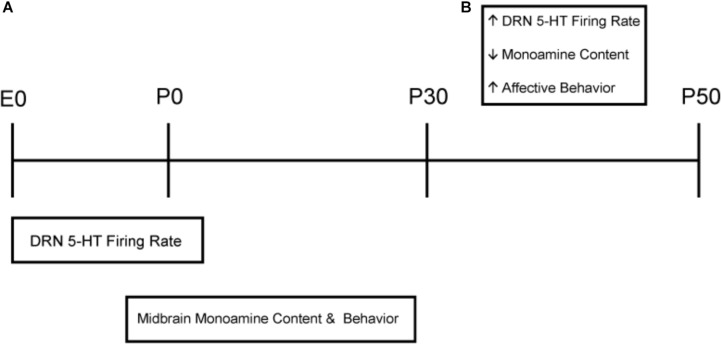
Sensitive periods impacted by Long photoperiod exposures and age related changes throughout development. **(A)** Long photoperiods program DRN 5-HT neuronal firing primarily during prenatal development, whereas exposure to Short photoperiods in gestation may extend this sensitive period into the early postnatal period. In addition, Long photoperiod exposures have both pre and postnatal origins impacting midbrain serotonergic content and affective behaviors later in life. **(B)** Age effects independent of photoperiod were observed in which adolescent (P30) mice demonstrated increased 5-HT neuronal firing rate in the DRN, no difference in 5-HT or 5-HIAA midbrain content and elevated levels of anxiety and depressive-like behavior compared to adult (P50) animals.

In this study we evaluated the effects of photoperiod exposure during prenatal and postnatal development on dorsal raphe serotonin neuron firing rate, midbrain serotonin signaling and depressive and anxiety-like behavior during stages of maturation representing adolescence and early adulthood in mice. Remarkably, we found that photoperiodic exposure during gestation *in utero* alone is sufficient to program the firing rate of serotonin neurons through adolescence and adulthood. Exposure to Long photoperiods limited to prenatal development programmed DRN neuron excitability equivalent to continuous exposure to Long photoperiods throughout development and maturation. Interestingly, prenatal exposure to Short photoperiods followed by exposure to Long photoperiods beginning at birth also increased the firing rate of serotonin neurons later in life. Taken together these results indicate that Long photoperiods experienced prenatally can program serotonin neurophysiology in an enduring fashion, and suggest that prenatal Short photoperiods may also extend the sensitive period for 5-HT neuron photoperiodic programing into the postnatal interval in mice (Figure 6A).

Our electrophysiology results also indicate photoperiod-independent changes in excitability of 5-HT dorsal raphe neurons as they mature from early adolescence to adulthood (Figure 6B). To this point, regardless of prior photoperiod, the firing rate of dorsal raphe serotonin neurons was substantially higher at P30 compared to P50 ages representing early adolescence and early adulthood, respectively, in mice (Hefner and Holmes, 2007). The elevated overall firing rate at P30 likely reflects a maturing state of the serotonergic system, consonant with observed changes in receptor sensitivity and 5-HT neuronal morphological development observed at that time (Rood et al., 2014). In addition, it has been shown that perinatal photoperiod can impact the suprachiasmatic nucleus (SCN) and circadian system (Ciarleglio et al., 2011a). Therefore, these changes in dorsal raphe 5-HT neuronal firing rate may be due to differential developmental photoperiod effects upstream of the DRN in the suprachiasmatic nucleus. Interestingly, the relative differences in excitability driven by prior photoperiods are expressed at both P30 and P50 suggesting that they are set in motion earlier.

While Long photoperiod exposure programs 5-HT neuron excitability prenatally, the increase in neuron excitability itself does not appear sufficient to fully program serotonin and 5-HIAA content. For example, mice exposed to Long photoperiods prenatally exhibit increased midbrain 5-HT levels in adolescence, but this does not carry through to adulthood, suggesting the necessity of a second “hit” by photoperiod postnatally to comprehensibly program serotonin circuit function. Likewise, postnatal exposure to Long photoperiods demonstrated only trend level increases in 5-HT at P30 and P50, a reduced effect compared to mice maintained on Long photoperiods throughout gestation, development and maturation (Green et al., 2015). Interestingly, 5-HIAA content was elevated for animals exposed to Long photoperiods postnatally by adulthood. Thus, midbrain serotonergic content appears to be programmed by sequential action first requiring prenatal programing of serotonin neuron excitability and then a second component of postnatal photoperiod exposure. These findings suggest that prenatal sensitive periods exist for sequential programming of the serotonin system and that photoperiod may then influence circuit level changes during postnatal development.

Recent epidemiological data indicates that the life-time risk of depression may be reduced by high amplitude changes in photoperiod during human gestation (Devore et al., 2018). Thus our current study focused on the developmental intervals during which high amplitude swings in photoperiod may program the serotonin system in mice as a potential model for the human findings. Future studies could extend this paradigm using different photoperiodic amplitudes (e.g., Long to Equinox) to examine explicitly the role of amplitude as well as timing in 5-HT programming.

We investigated when in development does exposure to Long photoperiods impact mood-related behavior later in life. Prior studies have demonstrated relationships between monoamine content in the striatum, hippocampus and frontal/cerebral cortex and depressive-like behavior utilizing the tail suspension test (Rodrigues et al., 2002; Cryan et al., 2004, 2005). Mice exposed to Long photoperiods postnatally exhibit reduced depressive-like behavior in the tail suspension test compared to mice exposed to Long photoperiods prenatally and Equinox mice. However, this group showed only trend level effects for reduction of anxiety-like behavior in the OFT, which was a reduced effect compared to our previous study of photoperiodic programing throughout prenatal and postnatal development (Green et al., 2015). This suggests that for full photoperiod programing of anxiety-like behavior prenatal exposure to Long photoperiod and the initial programing of serotonin neuron excitability is necessary.

Based on our results, photoperiod impacts serotonin neuron excitability, serotonin signaling and affective behavior during different developmental time windows in mice (Figure 6A). DRN neuronal firing rate is programmed mainly *prenatally*, while photoperiodic effects on serotonergic content and behavior have both pre and postnatal origins. This suggests that photoperiodic programming proceeds in a sequence, with programming of 5-HT neuronal excitability as the earliest step, followed by additional changes in the brain circuits mediating affective behaviors occurring later, in part, as a consequence of the altered 5-HT neuron excitability. In our study we observed that Long summer-like photoperiods consistently modulate serotonin function by elevating 5-HT neuronal firing rate and serotonergic content, and can be protective for mood-related behavior later in life. Only one other recent study has evaluated the effects of *prenatal* exposure alone to either Long or Short photoperiods and demonstrated that photoperiod does not impact sensorimotor gating via pre-pulse inhibition in adulthood (Takai et al., 2018), highlighting the need for future studies to evaluate the impact of photoperiod on behavior throughout development.

These differential sensitive periods for programming of 5-HT neuron excitability, for serotonergic signaling, and behavior were evaluated with our experimental paradigm in which animals were exposed to conflicting photoperiods before and after birth. This artificial manipulation of photoperiod is useful to investigate developmentally sensitive periods, however, in natural circumstances we would expect that the photoperiod experienced both prenatally and in early postnatal development would essentially be the same, given the brief intervals for gestation and maturation in mice compared to the rates of real world seasonal change. Thus, developing mice that experienced Long summer-like photoperiods in gestation would likely also experience Long photoperiods in early postnatal development. Therefore, the prenatal Long photoperiod programing of increased 5-HT neuron excitability observed in our L-S group, and the Long photoperiod postnatal effects of increased serotonin and 5-HIAA content along with reduced affective behaviors observed in our S-L group, would, in nature, normally proceed in sequence, resulting in positive concerted changes in neuronal activity, monoamines and affective behaviors. Indeed, this was previously observed when Long and Short photoperiod exposures spanned gestation, postnatal development and maturation (Green et al., 2015).

Mechanistically, increases in 5-HT neuronal firing rate may result in an increased demand for 5-HT synthesis in 5-HT neurons, which then manifests as increases of monoamine content in the midbrain. Our findings of differences in the time windows for programming between electrophysiology and serotonergic signaling along with behavior suggest roles for circuit level changes that will be of interest to address in the future. While we have investigated neuronal firing rate in the DRN, it will be critical to evaluate potential effects in connected brain regions downstream of the DRN such as the nucleus accumbens, ventral tegmental area and prefrontal cortex, which have also been consistently implicated in mood-related disorders (Krishnan and Nestler, 2008; Warden et al., 2012; Dölen et al., 2013).

In addition, melatonin signaling may be critical for the timing of programming. Previous work has shown that photoperiodic programing of DRN serotonin neurons during perinatal development is dependent on melatonin signaling through the MT1 melatonin receptor (Green et al., 2015). Interestingly, it has been demonstrated that prenatal melatonin signaling programs the rate of sexual maturation in male hamster pups in adolescence according to the photoperiod experienced *in utero* (Weaver et al., 1987) suggesting that photoperiodic programming of that system may be due to *in utero* maternal-fetal melatonin signaling (Weaver et al., 1987; Goldman, 2003). While melatonin receptors have not been demonstrated in the mouse dorsal raphe nucleus, melatonin receptors are present in the suprachiasmatic nucleus (SCN) in rodents as early as E18 in development (Davis, 1997) and the SCN is upstream of and indirectly projects to the dorsal raphe nucleus (Ciarleglio et al., 2011b). Therefore, it is plausible that the photoperiodic changes in DRN physiology during prenatal and perinatal development may be due to maternal-fetal melatonin signaling to the SCN, with projections to the DRN potentially driving the observed 5-HT neural changes. To this point, perinatal photoperiod has been shown to have enduring effects on the rhythmic properties of the SCN clock (Ciarleglio et al., 2011a). Future studies will need to be performed to more fully elucidate the neural underpinnings of these photoperiodic effects.

The prenatal programming of DRN 5-HT neurons via Long photoperiodic exposure we have observed here is likely due at least in part to maternal-fetal signaling, given the key involvement of melatonin, which is not synthesized by pups *in utero* or rhythmic in the early postnatal period (Tamarkin et al., 1980; Yellon et al., 1985; Davis, 1997; Goldman, 2003). Studies have demonstrated that the fetal brain is informed of and shaped by maternal signals indicating specific aspects of the expected neonatal environment, in particular, resources [maternal nutrition (Palmer et al., 2008; Bale et al., 2010) and stress (Schneider et al., 1999; Weinstock, 2001; Van den Bergh et al., 2005; Seckl and Holmes, 2007)]. Our results suggest that the fetal brain is also informed of and shaped by the external photoperiod, which may be another proxy for expected resources, with Long summer-like photoperiods indicating plentiful resources. In this case, biasing behaviors toward active exploration during early life, while biasing toward passive coping and conservation in resource limited Short winter-like photoperiods, may have advantages. Importantly, the effects of photoperiod in humans during maternal pregnancy and the risk for depression are just beginning to be evaluated (Devore et al., 2018). In this human epidemiological study it was demonstrated that photoperiodic exposure specifically during the second trimester of birth can result in a 13% reduction in the odds of depression for the offspring later in life (Devore et al., 2018). Thus, clinical evidence is beginning to demonstrate the photoperiodic effects during maternal pregnancy and the potential enduring effects this has for depression risk in the offspring in adulthood.

Our animal model may also provide valuable insight into photoperiodic mechanisms influencing affective disorders. Clinically, the incidence of mood disorders, specifically depression, is increased in early adolescence compared to early adulthood (National Institute of Mental Health [NIMH], 2015a,b), higher rates for depression have been observed in women compared to men (National Institute of Mental Health [NIMH], 2015b) and the prevalence for depression is highest in females during early adolescence with rates occurring at almost 20% (National Institute of Mental Health [NIMH], 2015a). In our study, depressive-like behavior was significantly elevated during adolescence in mice (P30), dramatically decreased in adulthood (P50) (Figure 5C), and showed a female bias (Supplementary Figure S2). Behavioral differences between photoperiods remained consistent across the developmental time points suggesting that early adolescence may be a susceptible time point in which depressive-like behaviors may be elevated in mice (McCormick and Green, 2013), as age dependent changes in mood related behaviors have been reported in mice (Hefner and Holmes, 2007; Mitchell et al., 2015). While drawing parallels between basic science and clinical studies has limits, the current findings can be further investigated to gain better understanding into the interplay between developmental photoperiod and the pathophysiology for mood disorders. In addition, these findings as well as recent human epidemiological work may suggest that light therapy during pregnancy could be a novel intervention with potential clinical impact in the development of affective disorders.

Overall, we suggest that serotonin function may be programmed by photoperiod sequentially, with differential sensitive periods for neuronal excitability, serotonergic signaling and behavioral outputs. Prenatal photoperiod can program dorsal raphe serotonin neuron excitability in an enduring fashion, while photoperiodic effects on midbrain serotonin levels and its’ metabolites, along with depressive and anxiety-like behaviors, requires additional postnatal photoperiod exposure, as would occur in natural circumstances. Importantly, Long photoperiod exposures during key sensitive periods in prenatal development program the DRN serotonin neurons initially and therefore may impact the development of affective disorders later in life. Evaluating environmental effects such as photoperiod during sensitive time periods throughout development may therefore provide critical insights into the underlying mechanisms and potential novel therapeutic treatments for mood-related disorders.

## Contribution on the Field Statement

Photoperiod or the duration of daylight directly impacts the serotonin system and has been consistently implicated in mood disorders. Rodent studies have shown that Long summer-like photoperiods experienced throughout development result in increased serotonin neuronal firing rates, elevated midbrain monoamine concentrations and decreased anxiety and depressive-like behavior. Importantly, recent human epidemiological work has demonstrated that high amplitude photoperiodic changes (i.e., large seasonal light transitions) experienced during the second trimester of pregnancy can result in a significant decrease in the risk of depression for the offspring later in life. We have now built upon these findings utilizing a mouse model that is melatonin-competent and a photoperiod paradigm of high amplitude changes to evaluate if photoperiods experienced either in prenatal or postnatal development impact dorsal raphe serotonin neurophysiology, signaling, and affective behavior in adolescence and adulthood. Remarkably, we found that Long photoperiods experienced during prenatal development can program adult serotonin neuron firing rate whereas Long photoperiods experienced mainly postnatally impact serotonin signaling and mood-related behaviors later in life. We suggest that photoperiodic programming may occur in sequential steps with serotonin neuronal programming occurring prenatally and with this step needed for full programming of circuit and behavioral-level changes occurring mainly during postnatal development.

## Data Availability

The datasets generated for this study are available on request to the corresponding author.

## Ethics Statement

All experiments performed were in accordance with the Vanderbilt University Institutional Animal Care and Use Committee and National Institutes of Health guidelines.

## Author Contributions

JS, NG, and NR conducted the experiments. JS, NG, and DM designed the experiments. JS and DM wrote the manuscript.

## Conflict of Interest Statement

The authors declare that the research was conducted in the absence of any commercial or financial relationships that could be construed as a potential conflict of interest.
