# Supplementary material for: Sequential Photoperiodic Programing of Serotonin Neurons, Signaling and Behaviors During Prenatal and Postnatal Development
Source: Front Neurosci. 2019 May 8;13:459. doi: 10.3389/fnins.2019.00459 (PMC6517556; doi:10.3389/fnins.2019.00459)
Supplement: Supplementary file 1 [file Data_Sheet_1.docx]

**Sequential photoperiodic programing of serotonin neurons, signaling and behaviors during prenatal and postnatal development**

**Supplementary Methods**

**Statistical Analysis: Monoamine Concentration and Behavior Experiments**

D’Agostino and Pearson normality tests were used and any mouse that was outside the normal distribution for any test was excluded from analysis (Tatem et al., 2014). This resulted in 1 mouse from the Eq at P30, 1 mouse from the Eq at P50, 2 mice from the L-S at P30, 2 mice from the L-S at P50, 2 mice from the S-L at P30 and 2 mice from the S-L at P50 groups being excluded. Overall, this resulted in 10 out of a total of 110 mice being excluded from behavioral studies. For behavioral studies, the final sample sizes were as follows: Eq at P30 (13 total mice: 5 males and 8 females), L-S at P30 (21 total mice: 7 males and 14 females), S-L at P30 (17 total mice: 7 males and 10 females), Eq at P50 (15 total mice: 8 males and 7 females), L-S at P50 (18 total mice: 10 males and 8 females) and S-L at P50 (16 total mice: 9 males and 7 females). In addition, 1 mouse from the S-L at P50 group was excluded from all monoamine analyses as it was outside the normal distribution based on a significantly low tissue level. This resulted in 1 out of a total of 76 mice being excluded from monoamine studies. For monoamine studies, the final sample sizes were as follows: Eq at P30 (12 total mice: 9 males and 3 females), L-S at P30 (13 total mice: 6 males and 7 females), S-L at P30 (13 total mice: 7 males and 6 females), Eq at P50 (14 total mice: 10 males and 4 females), L-S at P50 (12 total mice: 7 males and 5 females) and S-L at P50 (11 total mice: 5 males and 6 females).

**Supplementary Figures**

Supplementary Figure 1. Schematic diagram of multielectrode array recordings of DRN 5-HT cells. A) A representative cartoon of a perforated 6x10 multielectrode array (Multi Channel Systems) along with a bright field image of a DRN slice placed on the array. The diameter of the electrodes were 30 microns with 100 micron spacing in between each electrode (single black dot) resulting in the electrodes covering an area of 1200 microns below the cerebral aqueduct (white area at the top of the image) and 340 microns laterally on each side covering a total of 680 micron width. B) Representative average waveform of a 5-HT DRN cell depicted in yellow and a non-serotonergic cell in green. C) Representative spike trace and raster plot of a DRN 5-HT cell during initial recording and once 8OH-DPAT is applied to evaluate 5-HT1A suppression of firing rate. Note the drastic decrease in firing rate once 8OH-DPAT is applied via the depicted arrow. The cartoon utilized for Figure A) has been adapted from Multi Channel Systems, https://www.multichannelsystems.com/products/microelectrode-arrays/60mea50010ir-ti.

Supplementary Figure 2**.** Postnatal photoperiod results in age and sex dependent effects on the tail suspension test (TST). **A)** Analysis of TST across photoperiods comparing male and female mice. **B)** Analysis of TST across age comparing male and female mice. The significance levels are as follows: (* = p < 0.05, ** = p < 0.01, *** = p < 0.001, **** = p < 0.0001). **A)** A significant main effect of sex (p < 0.0001; F (1, 94) = 23.1) and a trend level main effect of photoperiod (p = 0.0598; F (2, 94) = 2.903) were observed for total time spent immobile in the TST. Holm-Sidak’s multiple comparison tests revealed significant differences in total time spent immobile for female compared to male mice for Eq (p = 0.0367), L-S (p = 0.0016) and S-L (p = 0.0141) photoperiods. **B)** A significant main effect of age (p < 0.0001; F (1, 96) = 38.25) and a significant main effect of sex (p < 0.0001; F (1, 96) = 21.4) were observed. Significant increases in time spent immobile were observed for females compared to males in adolescence (P30) (p = 0.0042) and in adulthood (P50) (p = 0.0010). In addition, significant Holms-Sidak’s multiple comparison age differences were found for males (p < 0.0001) and for females (p < 0.0001) such that adolescent (P30) animals demonstrated elevated levels of time spent immobile compared to adult (P50) animals.

Supplementary Figure 3**.** Postnatal Long photoperiod exposures reduces anxiety-like behavior utilizing the open field test. **A)** Analysis of thigmotaxis across photoperiods at P30, **B)** at P50 and **C)** within photoperiods during the P30 and P50 time points. The significance levels are as follows: (** = p < 0.01) using Holm-Sidak’s multiple comparison tests. **A)** Long photoperiod exposure during postnatal development resulted in trend level decreases of thigmotaxis (S-L vs. Eq, p = 0.0976, S-L vs. L-S, p = 0.0976) in adolescent (P30) and **B)** in adult (P50) mice (S-L vs. Eq, p = 0.0906). **C)** Holm-Sidak’s multiple comparison tests revealed within group differences for the L-S photoperiod (p = 0.0011) when comparing the P30 and P50 time points.
