# Supplementary figures and images for: Sequential Photoperiodic Programing of Serotonin Neurons, Signaling and Behaviors During Prenatal and Postnatal Development
Source: Front Neurosci. 2019 May 8;13:459. doi: 10.3389/fnins.2019.00459 (PMC6517556; doi:10.3389/fnins.2019.00459)

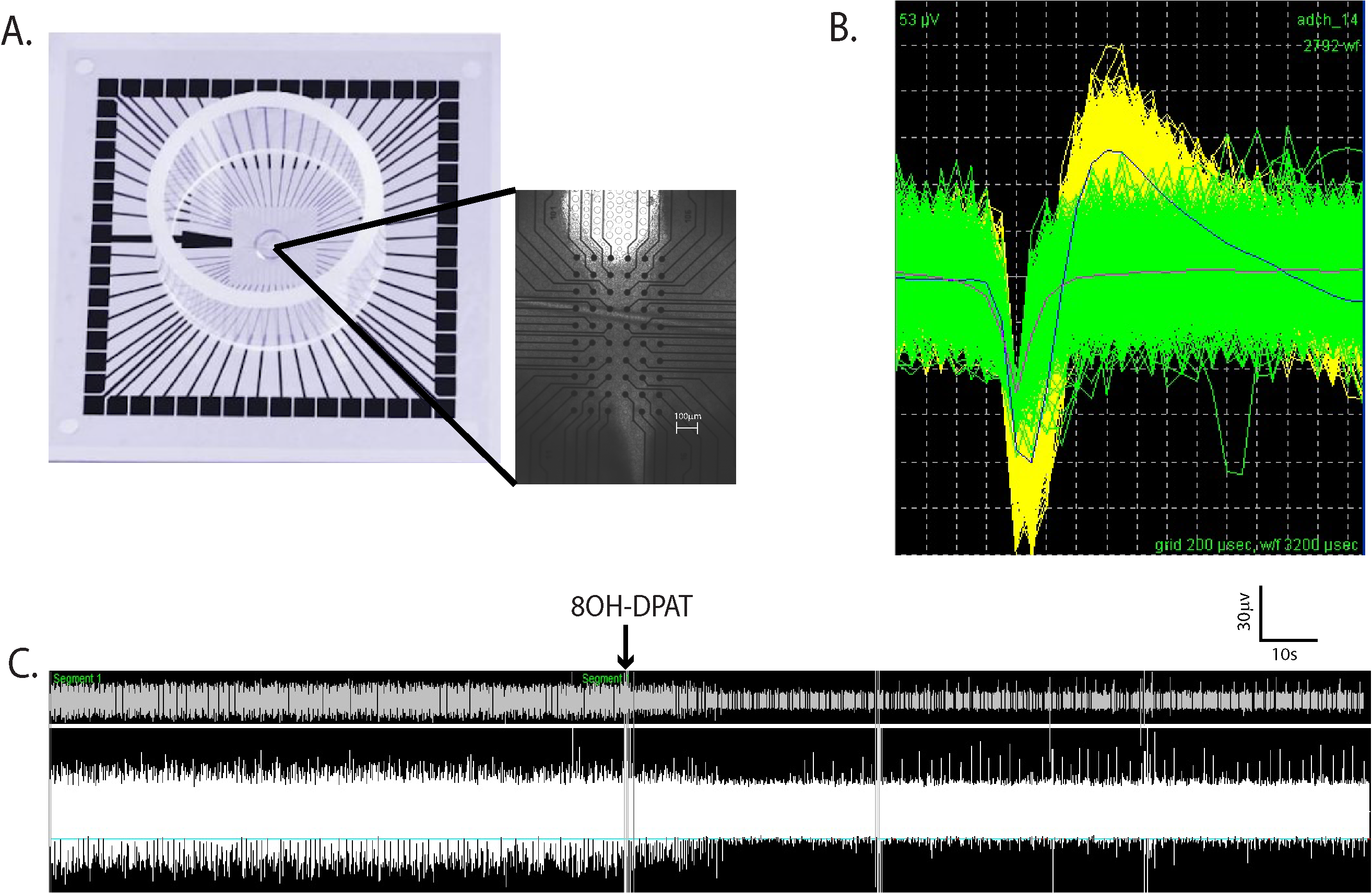

Supplement: Supplementary file 2 [file Image_1.TIF]

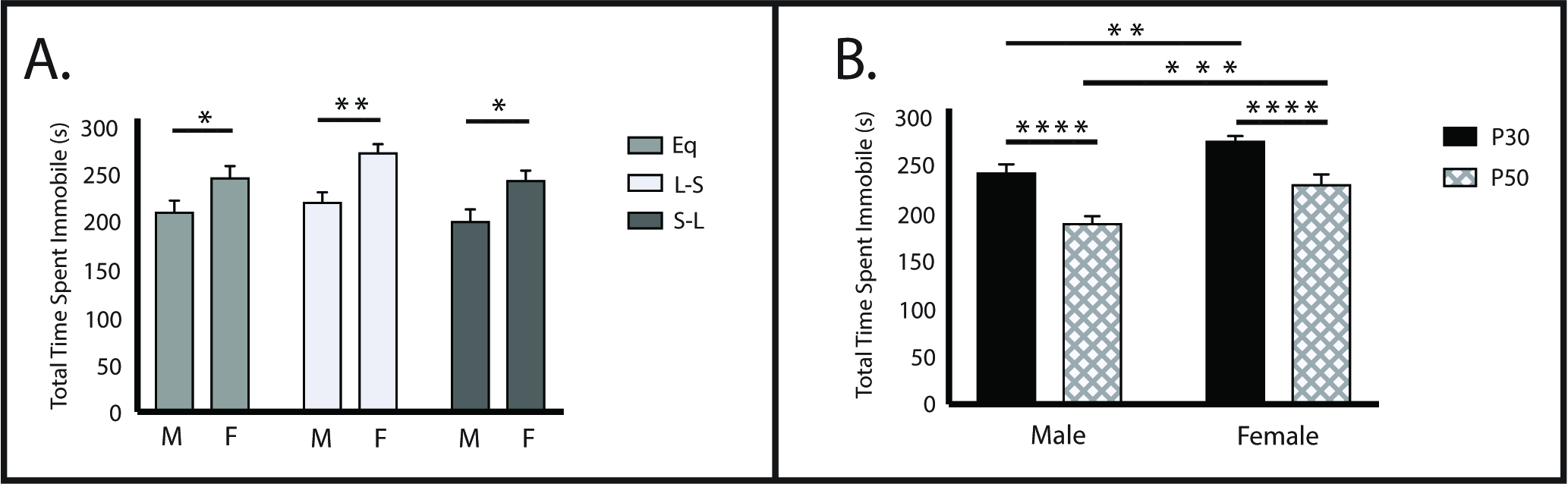

Supplement: Supplementary file 3 [file Image_2.TIF]

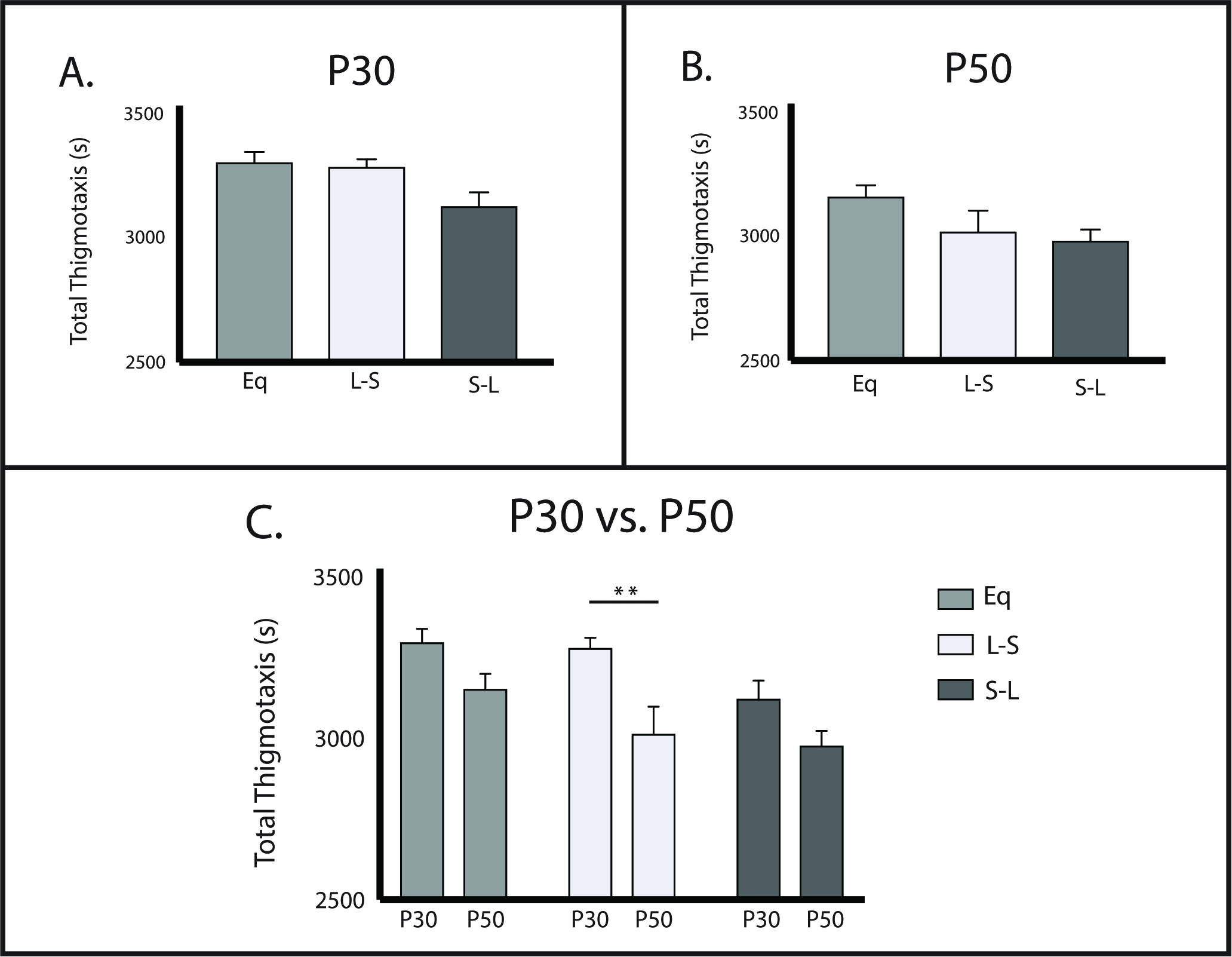

Supplement: Supplementary file 4 [file Image_3.TIF]
